# Networking experiences for promoting HIA implementation in Andalusia

**DOI:** 10.1093/eurpub/ckac129.103

**Published:** 2022-10-25

**Authors:** P Martin-Olmedo, L Moya Ruano, E Martin Verdugo, FJ Rodríguez Rasero, JL Martín-Jiménez, FJ Marchena

**Affiliations:** 1 EUPHA-HIA; 2 Public Health, Escuela Andaluza de Salud Pública, Granada, Spain; 3 Biosanitary Research Institute of Granada, IBs.Granada, Granada, Spain; 4 HIA Division, Consejería de Salud y Familias, Seville, Spain

## Abstract

The Health Impact Assessment (HIA) is a useful tool that allows policymakers and project developers to predict and control the consequences of their proposals on the health and equity of the affected population. The existence of a legislative framework for the institutionalization of HIA has been proposed as a critical factor in providing permanent rules and in legitimizing HIA within the decision-making process. In Andalusia (Southern Spain), the HIA is legally binding after the approval of the Public Health Act 16/2011 and Decree 169/2014, which establishes the HIA procedure in the Andalusian context. This new legal framework made necessary to promote capacity-building programs in HIA, but also to develop an online platform that would allow sharing experiences and knowledge among all those public health professionals involved in the review and approval of HIA reports submitted by the developers of proposals. These tasks were addressed by a leading group of the HIA network made up of officials from the Regional Ministry of Health and experts from the Andalusian School of Public Health (EASP). The platform, set up in 2018, integrates around 90 professionals from different administrative bodies according to the proposal's scope (local, regional), covering the great geographical dispersion of Andalusia, and allowing to evaluate more than 2400 proposals in 4 years. The platform also facilitates the traceability of files (both for improving internal management and in responding to external queries), and acts as a source of relevant evidence, supporting the work of professionals involved in HIA. The platform allows extracting data and generating figures, showing information on the number of files created and resolved by different periods, provinces, type of proposals, etc. This tool has been very useful for the continuous training of professionals involved in HIA in Andalusia, following a coaching approach.

**Speakers/Panellists:**

**Robert Otok**

ASPHER, Brussels, Belgium

